# The first report on internal transcribed spacer region-based characterization of microfilaria in Asian elephants (*Elephas maximus*) in Thailand

**DOI:** 10.14202/vetworld.2021.2260-2266

**Published:** 2021-08-28

**Authors:** Choenkwan Pabutta, Nuttapon Bangkaew, Pratthana Inthawong, Pannarai Mahadthai, Waleemas Jairak, Nantana Soda, Manakorn Sukmak, Supaphen Sripiboon

**Affiliations:** 1 Elephant Kingdom Project, Zoological Park Organization of Thailand, Tha Tum, Surin, Thailand; 2 Monitoring and Surveillance Center for Zoonotic Diseases in Wildlife and Exotic Animals, Mahidol University, Salaya, Nakhon Pathom, Thailand; 3 Center of Excellence in Elephant and Wildlife Research, Faculty of Veterinary Medicine, Chiang Mai University, Chiang Mai, Thailand; 4 Bureau of Conservation and Research, Zoological Park Organization of Thailand, Bang Sue, Bangkok, Thailand; 5 Kamphaeng Saen Veterinary Diagnostic Center, Faculty of Veterinary Medicine, Kasetsart University, Kamphaeng Saen, Nakhon Pathom, Thailand; 6 Department of Farm Resources and Production Medicine, Faculty of Veterinary Medicine, Kasetsart University, Kamphaeng Saen, Nakhon Pathom, Thailand; 7 Department of Large Animal and Wildlife Clinical Science, Faculty of Veterinary Medicine, Kasetsart University, Kamphaeng Saen, Nakhon Pathom, Thailand

**Keywords:** Asian elephant, genotype, internal transcribed spacer, microfilaria, Thailand

## Abstract

**Background and Aim:**

Filarial infections can significantly impact the health of both humans and animals. In elephants, filariasis has been associated with cutaneous dermatitis and skin nodules. However, molecular evidence for such infections is limited in Thailand. This study aimed to identify the morphological and molecular characteristics of microfilaria in captive Asian elephants in Thailand.

**Materials and Methods:**

Whole blood collected from the ear vein of 129 captive Asian elephants was hematologically analyzed, and the blood parasites were evaluated using three standard techniques: The microcapillary test, thin blood smears, and polymerase chain reaction (PCR).

**Results:**

Conventional PCR revealed that approximately 17% (22/129) of the sampled elephants were positive for microfilaria. Microscopy revealed that microfilariae are large, unsheathed, with extended nuclei, a short headspace, and a curved tail tapering at the end. Results of internal transcribed spacer region analysis show that the elephant microfilariae are closely related to *Onchocerca* spp. All of the elephants positive for microfilaria presented with neither skin lesion nor anemic signs. Microfilaria infection was not associated with age; however, microfilariae were more likely to be detected in male elephants due to differences in management systems.

**Conclusion:**

This is the first study to provide both morphological and molecular evidence of microfilaria in Thai elephants. There is an urgent need to investigate the long-term and large-scale effects of microfilaria on the health of elephants.

## Introduction

The Asian elephant (*Elephas maximus*) has been listed as endangered by the IUCN, and its current populations continue to decline. The major threats to this animal include the loss and fragmentation of its habitat and poaching [1]. The largest Asian elephant population is in India (~30,000), and other relatively large populations are in Sri Lanka (~5800), Malaysia (~4000), Myanmar (~2000-4000), and Thailand (~3000-4000) [1]. Elephants play an important role in the ecosystem [2], and in Thailand, this animal is also culturally and economically significant [3,4]. Efforts to conserve elephants focus on changing environmental factors and habitat destruction; however, diseases are also of increasing concern. Knowledge gaps exist in the health and disease management of elephants in Thailand; for example, disease status, prevention protocols, and monitoring programs all need further studies.

Blood parasite infections are a source of concern to the medical and veterinary communities. Such infections cause significant impacts on health; for example, they can cause elephantiasis and river blindness in humans [5,6], heartworm disease in dogs [7], trypanosomiasis in domestic animals [8], and babesiosis in cattle [9]. In elephants, the blood parasite infections that cause the most concern are trypanosomiasis, babesiosis, and filariasis, all of which cause anemia and impair body fitness [10]. This study mainly focuses on filarial infection.

At the early larval stage, filarial worms are called microfilaria, and they are found in the bloodstream or lymphatic system of the host. Microfilaria may be transmitted to other hosts by blood-feeding insects (hematophagous arthropods vectors) [11]. The parasite develops in both vector and host [11], and the adult worm may reside in different target organs, depending on the species [12]. The three main diagnostic methods for filariasis are as follows: (1) Traditional morphological analysis, targeting both adult and larval stages; (2) collecting biological data, including host preference, type of vector, and geographical distribution; and (3) molecular analysis [13]. Traditional morphological analysis requires expertise and is prone to misdiagnosis; thus, molecular techniques have become widely used to identify filaria [12-15]. Because molecular techniques provide accurate information and are highly sensitive compared to other methods, they are often used for epidemiological studies [12]. Among several polymerase chain reaction (PCR)-based methods for filaria detection and identification [12], the analysis of variations within the internal transcribed spacer (ITS) region has been suggested to be an informative and relatively simple method to obtain general genetic data [12,16]. Filariasis occurs in both African and Asian elephants, and infections generally involve the presence of skin nodules (1-2 cm in diameter) at the abdomen and toenail bed [10,17]. Several filaria species have been identified in elephants. For example, *Loxodontofilaria loxodontis* and *Loxodontofilaria gossi* occur in African elephants, while *Loxodontofilaria asiatica*, *Indofilaria parabiramani*, and *Stephanofilaria* spp. occur in Asian elephants [10,17]. However, none of these previous studies have provided any genetic data.

This study aimed to identify microfilaria in captive Asian elephants in Thailand based on the analysis of the ITS region. We also characterized the microfilaria morphologically, through microscopic examination. Because this blood parasite has been associated with anemia in elephants [10], we generated blood profiles and recorded the clinical appearance of filaria-positive elephants. In addition, we evaluated the possible risk factors (i.e., sex, age class, and location) associated with microfilaria infection to develop appropriate prevention and monitoring protocols.

## Materials and Methods

### Ethical approval

This study was a part of the routine health check at Elephant Kingdom Project, Zoological Park Organization of Thailand, for which ethical approval was not obligatory.

### Study period, area, and animals

The study was conducted from September 1 to 5, 2020. This study was part of the annual routine health check at the Elephant Kingdom Project (EKP) managed by the Zoological Park Organization of Thailand, located at Surin Province, Northeast Thailand. The EKP comprises approximately 1000 acres and contains approximately 200 captive Asian elephants (*E. maximus*). The elephants are dewormed using febentel (Rintal® Bolus, Bayer Thai Co., Ltd., Thailand; 6 mg/kg, PO) every 4-6 months, and they receive a complete health examination annually. Any emergency health issues are treated under the supervision of on-site veterinarians.

### Sample collection

Veterinarians physically examined a total of 195 elephants as part of their routine annual health check. All health issues were recorded and blood samples were collected from the elephants using positive reinforcement training techniques or physical restraint. Physical restraint involved the use of a rope and bar to tie one of the elephant’s legs to minimize movement. Blood samples were collected from the other side of the bar by a skilled veterinarian to prevent any injuries and minimize sampling time. All the elephants included in this study were well trained. However, if an elephant was reluctant to participate or cooperate during sample collection, then that elephant was excluded from the study. Whole blood and serum samples were collected using the following protocol:

A minimum of 5 mL of blood was collected from the auricular ear vein. The sample was aliquoted into an ethylenediaminetetraacetic acid-coated tube and a plain tube. All samples were stored at 4°C during transportation. Hematological and microscopic examinations were conducted within 6 h after samples were collected. The remaining whole blood samples were then kept at −20°C and submitted to the Faculty of Veterinary Medicine, Kasetsart University (Kamphaeng Sean Campus) to test for blood parasites using molecular techniques.

### Laboratory analyses

#### Blood profile

Hematological analyses were carried out by skilled technicians. Analyses included total red blood cell count, total white blood cell count, differentiation number of white blood cells, and pack cell volume. Serum samples were submitted to the Veterinary Research and Development Center, Surin Province, to evaluate the following: Alanine transaminase, aspartate transaminase (AST), alkaline phosphatase, blood urea nitrogen, creatinine, bilirubin, creatine kinase, and total plasma protein levels.

### Microscopic examination of blood parasites

Whole blood was examined under the microscope using two standard techniques: (1) The microcapillary test (MCT) and (2) the thin blood smear (TBS) technique [18]. In the MCT, microhematocrit tubes were filled with blood samples; then the tube was sealed at one end with silicone. The tube was centrifuged at 8000 x *g* for 5 min, then the buffy coat layer was examined under a light microscope to detect blood parasite movement. The TBS method followed the standard protocol, which uses a light microscope to examine Wright-stained blood smears. In addition, random microfilaria-positive samples were analyzed by Knott’s modified test to determine the abundance and evaluate the morphological features of microfilaria [18,19]. Briefly, 0.5 mL of whole blood samples were mixed with 4.5 mL of 2% formalin solution, and then the mixture was centrifuged at 3000 × *g* for 5 min. The pellet was stained with 0.1% methylene blue, then the microfilariae were observed under a light microscope. All the samples were processed within 6 h of collection.

### Molecular analysis for blood parasites

All blood samples were tested for blood parasites using PCR. DNA was extracted from 100 μL of whole blood using the modified phenol-chloroform method [20]. Four primer pairs were used to detect the blood parasites, including *Babesia/Theileria* spp. [21], *Trypanosome* spp. (Wajjwalku: unpublished data), and *Filaria* spp. [22,23]. In addition, we analyzed ITS region to determine the species of microfilaria. Primers UNI-1R and FIL-1F [22] were used to amplify the complete ITS1 region (18s, ITS1, and 5.8s), while primers DIDR-F1 and DIDR-R1 [23] were used to amplify the 5.8s, ITS2, and 28s regions. The details of each primer set are listed in Table-1 [21-23]. Platinum™ Hot Start PCR Master Mix (Thermo Fisher, USA) was used in each PCR reaction according to the manufacturer’s protocol. Sterile water was used instead of a DNA template as a negative control to check for any contamination. DNA extracted from *Babesia bigemina*, *Trypanosoma evansi*, and *Microfilaria immitis* were used as positive controls for each primer for blood parasite infection (Table-1). All PCR products of the correct size were purified using a FavorPrep™ GEL/PCR Purification Kit (Favorgen Biotech Co., Taiwan) according to the manufacturer’s instructions. The purified amplicons were sequenced using a BigDye^®^ Cycle Sequencing Kit (Applied Biosystems, USA) and an ABI PRISM 3130 Automated DNA Sequencer (Ibis Biosciences, USA). Sequences were analyzed using BioEdit^®^ (Ibis Biosciences) and compared to database sequences through BLASTn searches. The reference sequences of the closest filarial matches were downloaded from NCBI and, together with the sequence generated in this study, aligned using MUSCLE in MEGAX [24] with standard settings and trimmed. Maximum likelihood (ML) trees were constructed to verify the phylogenetic relationships between microfilaria in elephants. Before constructing the ML tree, a best-fit substitution model was selected based on MEGAX recommendations. We used the Tamura 3-parameter model with a discrete gamma distribution (T92+G) to generate a phylogenetic tree with a thousand bootstrap replicates. The mean distance between groups was calculated by pairwise distance analysis using MEGAX with *p*-distance substitution model.

**Table-1:** List of primers used in this study.

Target	Primer	Temp. (°C)	Expected size (bp)	Reference
*Babesia/Theileria* spp. (18s)	BAB1w: 5’-GAACCTGGTTGATCCTGCCAG-3’ BAB2w: 5’ GATCCTTCTGCAGGTTCACCTA-3’	54	1600	[21]
*Trypanosome* spp. (18s)	Tryp1w: 5’-GCGCATGGCTCATTACATCAGACGTAA-3’ Tryp2w: 5’-TCTTGATTGAGGAAGGTATCCTTGAAG-3’	54	1100	U/D
*Filaria* spp. (18s-ITS1-5.8s)	UNI-1R: 5’-CGCAGCTAGCTGCGTTCTTCATCG-3’ FIL-1F: 5’-GTGCTGTAACCATTACCGAAAGG-3’	60	712-771a	[22]
*Filaria* spp. (5.8-ITS2-28s)	DLDR-F1: 5’-AGTGCGAATTGCAGACGCATTGAG-3’ DLDR-R1: 5’-AGCGGGTAATCACGACTGAGTTGA-3’	60	430-664a	[23]

Ta = Annealing temperature, U/D = Unpublished data, ^a^size depending on the species of filaria

### Statistical analysis

The statistical relationships, including odds ratios and p-values, between sex, age class, blood values, and microfilaria infection were calculated using the Epitools program [25]. The level of significance was set at p=0.05. The statistical significances were determined using Chi-square tests when categories were >5 and with Fisher’s exact two-tailed tests when any one category was <5.

## Results

Of the 195 captive Asian elephants that were examined, samples were collected from 129, which were composed of 55 males and 74 females. All of the following data refer to the sampled elephants [26]. None of the elephants were less than 1 year old; 12 elephants were juvenile (1-5 years), 38 were sub-adult (5-15 years), 72 were adult (15-50 years), and 7 were >50 years old. All of the elephants were healthy and without any major health issues at the time of sampling. Hematological analysis revealed high levels of variation individually. Packed cell volume (PCV) values >40 (i.e., high PCVs) were found in 39% of the elephants, which is possibly due to dehydration. All of the elephants’ red blood cell counts were within the normal range (2.5-5×10^6^ cells/μL). Leukocytosis (white blood cell >18,000 cells/μL) was detected in 18% of the elephants. Most of the elephants’ blood chemistry profiles were in the normal range, although 5% of the elephants had elevated AST levels (higher than 35 IU/L).

Microfilariae were detected in 15 of the elephants (11.6%). PCR analysis did not detect any *Babesia* spp., *Theileria* spp., or *Trypanosome* spp.; however, 22 samples (17.05%) were positive for microfilaria. Of the 22 microfilaria-positive elephants, 16 were female and 6 males; their ages ranged from 3 to 34 years (the median age was 16 years). Microfilariae were more likely to be detected in adult elephants (15-50 years), and no significant association was found with other age classes (OR=1.48; 95% CI: 0.57-3.83; p=0.42). In addition, male elephants were more likely to be microfilaria positive than females (OR=4.65; 95% CI: 1.68-12.86; p<0.05). None of the microfilaria-positive elephants presented with signs of anemia. Only 14.7% (5/22) of the elephants in this group had elevated levels of eosinophils (more than 1000 cells/μL). No significant association was found between eosinophil count and the microfilaria-negative group (OR=OR=0.79; 95% CI: 0.27-2.34; p=0.79).

### Morphology of microfilaria in Asian elephants

**Figure-1:**
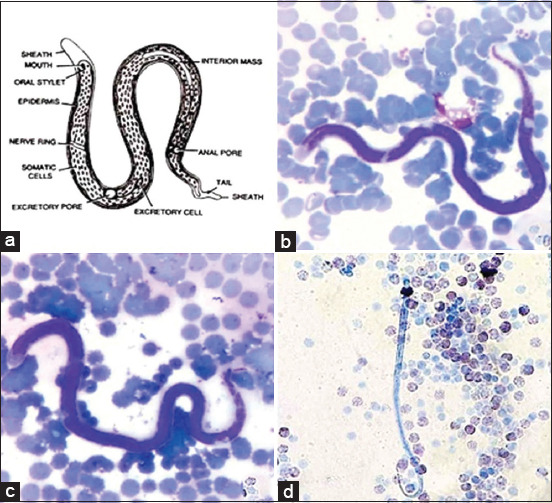
The morphological characterizations of microfilaria were revealed under light microscope using thin blood smear (b and c) and modified knotted (d) methods, comparing to the reference (a).

Microscopic analysis reveals that the microfilariae are large and unsheathed, with extended nuclei, a short headspace and a curved tail tapering to the end (Figure-1). The microfilariae have a body length of 270-300 μm and a width of 6-8 μm. The nerve ring, measuring 2-3 μm, is prominent, occupying 25% of the anterior end of the body. The excretory and anal pores have similar diameters of 4-6 μm, and are located 35% and 80% from the anterior end, respectively. Based on blood counts, microfilariae represented a mild infection, with only 1-2 microfilariae found in the buffy coat and TBSs. Moreover, we detected no adult worms in the subcutaneous tissue of the elephants.

### Molecular identification and phylogenetic analysis

**Figure-2:**
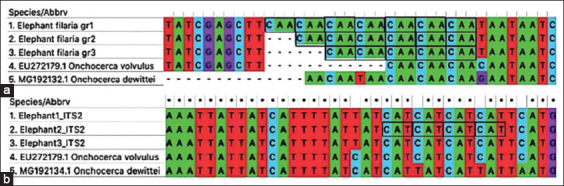
(a) The alignment of ITS1 region showed the variation of short repetitive DNA sequences (CAA) of elephant microfilaria found in this study. (b) The alignment of ITS2 region showed the variation of CAT repeats among different species of filaria.

**Figure-3:**
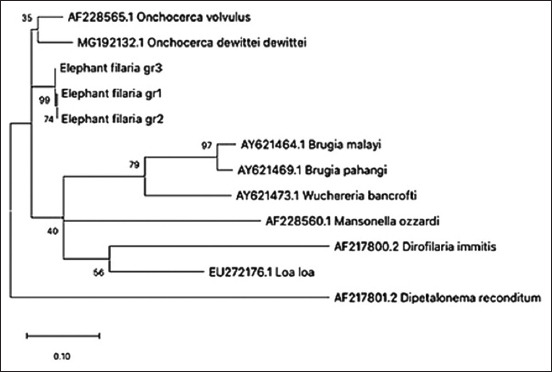
Phylogenetic tree based on ITS-1 gene sequence from filarial nematode specimens. The total length of the alignment is 437 bp. The topology was inferred using Maximum likelihood. Nodes are associated with bootstrap values based on 1000 replicates. Bootstrap support values <70 are not shown. The scale bar indicated the number of nucleotide substitutions. Elephant filaria gr1 refers to isolate ThE53 (GenBank accession no. MW001163), elephant filaria gr2 refers to isolate ThE47 (GenBank accession no. MW001164), and elephant filaria gr3 refers to isolate ThE44 (GenBank accession no. MW001165).

The length of the ITS1 region of elephant microfilaria ranged from 431 to 437 bp (GenBank Accession No. MW001163-65) and contained 5-7 short repetitive DNA sequences (CAA) within positions 9-30, causing the variation in this region’s length (Figure-2a). The majority of the ITS1 sequences contained seven CAA repeats, and the intraspecific *p*-distance between sequences of ITS1 was less than 1%. Due to the insufficient amount of extracted DNA, we were able to sequence the ITS2 regions from only three positive samples. We detected no variation in the length of ITS2 sequences, which were 364 bp long. Based on comparisons with database sequences, approximately 20 bp was missing at the 5′ end of ITS2 due to error at the sequencing process. Therefore, the length of the ITS2 region should be 384 bp (GenBank Accession No. MW001162). Similar to ITS1, ITS2 also contained short repeats (CAT), which were located toward to 5′ end. Different numbers of CAT repeats were found in different species of filaria. Our samples had four CAT repeats, while *Onchocerca volvulus* and *Onchocerca dewittei* contain five and one repeats, respectively (Figure-2b). Both ITS1 and ITS2 were A-T rich, with G-C contents of 25% and 19% in ITS1 and ITS2, respectively. The sequences of the ITS regions were closely related to reference sequences of *Onchocerca* spp., which can be seen as a clear cluster with high bootstrap support values in Figure-3.

## Discussion

Blood parasite infections are a most concerning issue in veterinary practice because they often impair health [12,27]. *Trypanosome* spp. that have been previously reported in captive Asian elephants in Thailand [28-30] cause severe lethargy and exercise intolerance. However, to the best of our knowledge, this study is the first to provide both morphological and molecular data regarding filariasis in captive Asian elephants in Thailand.

A previous study reported that 87% of adults and 31% of young African elephants were positive for filariasis [31], while our study found no association with any age class in a herd with 17% microfilaria infection. However, microfilariae were more prevalent in male elephants, which are possibly due to the management system of male elephants in Thailand. Because of their aggressive behavior, male elephants generally live in confined areas and are thus more likely to encounter a vector. Although none of the microfilaria-positive elephants lived in the same enclosure, we observed elephants from different enclosures occasionally encountering one another during daily activities. Thus, studying the epidemiology of microfilaria in elephants should include investigating the presence of the parasite in vectors and long-term monitoring of other animals that live in the same enclosure as a microfilaria-positive elephant.

The levels of microfilariae in the bloodstream may fluctuate throughout the day [10]. Thus, selecting an appropriate sampling time is important to enhance the chance of detecting parasites. The highest number of microfilariae is often detected between 21:00 and 03:00 [10,31]. However, we were unable to collect our samples during these times. Due to safety reasons, the samples were collected between 10.00 and 16.00.

The criteria for morphological identification of microfilariae include their size, sheath, and nuclear column [18]. The elephant microfilariae are unsheathed with extended nuclei; and they are considered large (270-300 mm in length and 6-8 mm in width), with dimensions that exceed those in a previous report (Table-2) [17,31]. Species identification based on morphology also requires analysis of the adult stage filaria. Adult filaria in elephants has been associated with skin nodules; however, our study did not find skin nodules in the microfilaria-positive group. Nevertheless, any elephant skin lesion should be noted and sampled (both blood and tissue) for further analysis.

**Table-2:** Host species, demographical location, and size of microfilaria found in this study, comparing to the previous reports [17,31].

	Our study	*L. asiatica*	*L. loxodontis*	*L. gossi*	*L. caprini*	*L. hippopotami*
Host	*Elephas maximus*	*Elephas maximus*	*Loxodonta africana*	*Loxodonta africana*	*Naemorhedus crispus*	*Hippopotamus amphibius*
Country	Thailand	Myanmar	Zaire	Tanganyika	Japan	South Africa
Body length	270-300	248-252	180-200	275	123-125	355-380
Body width	6-8	5.5	6.5-7.0	-	6-8	9-10

*L. loxodontis: Loxodontofilaria loxodontis, L. gossi: Loxodontofilaria gossi, L. asiatica: Loxodontofilaria asiatica, L. caprini: Loxodontofilaria caprini*

Molecular approaches can increase the sensitivity of microfilaria detection. Here, conventional PCR resulted in 5.4% increase in detection of microfilaria. In addition, ITS region-based analysis also provided genetic information that will be useful for disease prevention and epidemiological studies. Although the filaria, *L. asiatica*, has been reported in Asian elephants in Myanmar [10,31], its DNA sequence has not been deposited in any of the molecular databases. The only publicly available sequence information for *Loxodontofilaria* spp. was that of *Loxodontofilaria caprini*, which was originally found in a Japanese serow (*Naemorhedus crispus*) subcutaneous tissue [17]. However, the available genetic data for *L. caprini* do not include the sequence of its ITS region. Our analysis indicates that the elephant microfilaria is closely related to *Onchocerca* spp., a finding that was similar to those from a previous study showing that *L. caprini* is most closely related to *O. dewittei japonica* based on the *12*S, *18*S, and *28S rRN*A genes, and the *cox*I, *rbp*1, *hsp7*0, and *myoHC* genes [14].

All microfilaria-positive elephants were physically healthy and their blood profiles and serum blood chemistry were mostly within normal ranges. The exception comprised approximately 15% of this positive group that had elevated eosinophil levels. Previous microfilaria infections in elephants have been associated with skin nodules or hemorrhagic dermatitis [10,31]. We observed no skin lesions in infected elephants, which are consistent with the absence of health impacts on microfilaria-infected elephants. Nevertheless, long-term monitoring of infected elephants is required. Zoonotic filariasis has been reported in many parts of the world [32]. Although cross-species transmission of filaria from elephant to human has not yet been reported [31], further study is needed on the potential for cross-species disease transmission, considering the close relationship between captive elephants and humans in Thai culture.

## Conclusion

Relying on traditional morphological criteria to identify filaria infection may lead to species misdiagnosis. More reliable diagnoses can be achieved using molecular analysis, which also provides information on evolution, transmission, and epidemiology. Here, we characterized microfilaria from Asian elephants in Thailand using both morphological and molecular methods. The microfilaria-positive elephants were generally healthy, with no signs of anemia, skin nodules, or dermatitis. The microfilariae were large, unsheathed, with extended nuclei, short headspace, and a curved tail tapering to the end. Analysis of the ITS region indicates that they are closely related to *Onchocerca* spp. Although microfilaria in elephants seems to have minimal impacts on health, we recommend further long-term monitoring of clinical signs and blood indices in elephants, as well as vector and other animal surveillance.

## Authors’ Contributions

CP and SS: Planned the study design, analyzed, and drafted the manuscript. CP, NB, and PI: Collected the sample and data. PM and WJ: Analyzed the hematological and morphological results. NS and MS: Analyzed the molecular results. CP, SS, WJ, and MS: Reviewed the manuscript. SS: Supervised the research. All authors read and approved the final manuscript.
